# Graves’ Disease: Is It Time for Targeted Therapy? A Narrative Review

**DOI:** 10.3390/medicina61030500

**Published:** 2025-03-13

**Authors:** Nicola Viola, Alessandro Colleo, Mauro Casula, Chiara Mura, Francesco Boi, Giulia Lanzolla

**Affiliations:** 1 Endocrinology Unit, Department of Clinical and Experimental Medicine, University of Pisa, University Hospital of Pisa, 56100 Pisa, Italy; nicola.viola@phd.unipi.it (N.V.); maurocasula4@gmail.com (M.C.); 2 Endocrinology Unit, Department of Medical Science and Public Health, University of Cagliari, University Hospital of Cagliari, 09124 Cagliari, Italy; alecolleo@gmail.com (A.C.); chiara.mura@unica.it (C.M.); francesco.boi@unica.it (F.B.)

**Keywords:** Graves’ disease, thyroid autoimmunity, thyroid diseases, hyperthyroidism, targeted therapy

## Abstract

Current therapies for Graves’ disease (GD) primarily aim to manage hyperthyroidism through synthetic antithyroid drugs, radioiodine, or surgery. However, these approaches are often limited by their incomplete efficacy and the risk of inducing hypothyroidism. The latest advances in understanding the autoimmune mechanisms driving GD have paved the way for novel therapies targeting the thyrotropin receptor (TSH-R) or immune pathways. Overall, key targets include cluster of differentiation 20 (CD20), cluster of differentiation 40 (CD40), protein tyrosine phosphatase non-receptor type 22 (PTPN22), cytotoxic T lymphocyte antigen-4 (CTLA-4), B cell-activating factor (BAFF), and the Fc receptor-like protein 3 (FcRL3). Recent preclinical studies and clinical trials testing targeted therapies have shown promising results in terms of efficacy and safety. Here, we present a narrative review of the literature on emerging therapeutic approaches for GD that are currently under investigation.

## 1. Introduction

Graves’ disease (GD) is an autoimmune disease which arises from a complex interplay of pathogenic factors, with hyperthyroidism primarily driven by autoantibodies that stimulate the thyrotropin receptor (TSH-R) on thyroid epithelial cells [1].

GD is more common in women than in men, with a female-to-male ratio of ~10:1. Even though it can occur at any age, the average age of onset is between 30 and 50 years [2]. Although global geographic data remain poorly defined, its prevalence appears to be highest in Asia and lowest in sub-Saharan Africa [3].

GD is characterized by excessive production of thyroid hormones, which affects multiple systems and commonly causes tremors, heat intolerance, weight loss, breath shortness, insomnia, irritability, goiters, and cardiovascular issues such as tachycardia and atrial fibrillation [4]. A large cohort study including 500.000 people followed for over eight years reported a 13% cumulative incidence of atrial fibrillation among patients with thyrotoxicosis aged >65 years [5]. In addition, patients with GD can experience extrathyroidal manifestations, including Graves’ orbitopathy (GO), pretibial myxedema, and acropachy [6]. While pretibial myxedema and acropachy are less common, GO occurs in about 30% of patients, presenting with a clinical parade ranging from periorbital edema to diplopia and, in severe cases, vision loss [4,7]. According to the 2016 American Thyroid Association (ATA) Guidelines and the 2018 European Thyroid Association (ETA) statement, the diagnosis of GD relies on clinical, biochemical, and instrumental investigations [8,9].

Current therapeutic strategies restore normal thyroid hormone levels without acting on the causative molecular mechanisms of the disease. Recent advances in targeted therapies hold promise for revolutionizing GD management. In this narrative review, we explore key aspects of GD, with a particular focus on available and emerging treatments. We conducted a PubMed search with the following keywords: Graves’ disease, or GD, and genetic, or epigenetic, or gene polymorphism, or novel molecular mechanisms, or new treatments, or new therapies, or novel approaches, targeted therapies, or monoclonal antibodies. Only studies published in peer-reviewed journals were chosen. Non-English publications, case reports, letters to the editor, and conference abstracts were excluded.

## 2. Pathogenesis and Molecular Mechanisms

### 2.1. TSH-R Autoantibodies and Immune Tolerance Escape

Loss of self-tolerance allows thyroid-stimulating hormone receptor (TSH-R) peptides to be recognized by antigen-presenting cells (APCs) and presented with major histocompatibility complex (MHC) proteins. This process activates T cells, which then stimulate B cells through T-cell receptor (TCR)–MHC interactions and CD40-CD40L binding. Activated B cells differentiate into plasma cells that produce TSH-R antibodies (TRAbs), driving thyroid cell proliferation and hormone secretion, ultimately causing hyperthyroidism [10,11]. TSH-R-stimulating antibodies (TSAbs) target a conformational and discontinuous epitope in the amino-terminal region of the extracellular domain of the TSH-R [12]. Upon binding, TSAbs activate intracellular G proteins linked to the TSH-R, initiating the transcription of genes responsible for thyroid hormone production, including those encoding thyroid hormone-producing enzymes, Tg, and the sodium iodide symporter. This process occurs via the phospholipase-C and cyclic AMP pathways, driving thyrocyte proliferation, secretion, and the synthesis of thyroid hormones [13], resulting in excessive thyroid hormone production, which underpins the clinical manifestations of GD.

Sensitive immunoassays detect TSH-R antibodies (TRAbs) in nearly 95% of patients with Graves’ disease (GD) [14]. Anti-thyroperoxidase antibodies (AbTPO) can be found in approximately 70–80% of patients, though they are neither diagnostic nor predictive of prognosis [15]. In addition to TSAb, circulating antibodies inhibiting TSH-R signaling can occur, thereby lowering the effectiveness of stimulating antibodies. The erratic hyperthyroidism and the occurrence of hypothyroidism occasionally observed in GD patients can be partially attributed to the coexistence of these blocking antibodies alongside circulating TSAb [16].

Recent advances in single-cell RNA sequencing (scRNA-seq) have unveiled novel insights into immune dysregulation in GD, particularly in peripheral blood mononuclear cells (PBMCs) [17]. A recent study analyzing PBMCs from GD patients and healthy controls revealed significant alterations in immune cell composition and function [17]. Notably, GD patients exhibited an increased proportion of CD16+ natural killer (NK) cells and memory subsets within T and B lymphocytes, accompanied by heightened inflammatory activity. Furthermore, impaired intercellular communication was observed, particularly a reduction in NK cell interactions with CD4+ T cells, monocytes, and B cells, mediated by downregulated inhibitory killer-cell immunoglobulin-like receptors (KIR). These findings suggest that disruptions in immune cell crosstalk contribute to the loss of immune tolerance in GD, highlighting new potential targets for therapeutic intervention. Interestingly, a notable increase in the proportion of memory B cells within the PBMCs of GD patients was observed, indicating heightened antigen exposure and more active humoral immunity. Furthermore, transcription factors such as Transcription factor Spi-B and the Kruppel-like factor 10 axis (KLF10) displayed increased activity in memory B cells. Previous studies have shown that SPIB can upregulate anti-apoptotic and autophagy-related genes, thereby promoting the survival and formation of memory B cells. In contrast, KLF10 is known to enhance immune and inflammatory responses. Additionally, there was a significant upregulation of genes associated with B-cell activation and immune response-activating signaling pathways in B cells from PBMCs of GD patients compared to healthy subjects. Further experimental validation is needed to clarify whether these genes directly contribute to the disruption of self-tolerance and the pathogenesis of GD [17].

As is known, B cells and T cells play a pivotal role in the pathogenesis of autoimmune disease, including GD [11]. However, recent research has highlighted the crucial role of dendritic cells (DCs) in GD pathogenesis, particularly in shaping the autoimmune response [18]. DC dysfunction may contribute to the breakdown of immune tolerance, leading to the activation of autoreactive T and B cells. In an effort to restore immune tolerance, novel therapeutic strategies have focused on targeting DCs. A recent study developed polylactic-co-glycolic acid (PLGA) nanoparticles encapsulating the TSH-R autoantigen and the immune tolerance inducer rapamycin. These nanoparticles were efficiently taken up by DCs, inhibited their maturation, modulated cytokine release, and promoted regulatory T-cell (Treg) expansion, ultimately preventing and ameliorating disease in a mouse model, without toxicity [19]. These findings suggest that DC-targeted therapies may offer a promising new approach for inducing immune tolerance in GD.

### 2.2. Genetic and Environmental Risk Factors for GD

Although the immune tolerance escape and the presence of TSH-R antibodies are considered key pathogenic events in the disease, the etiopathogenesis of GD is multifactorial, involving a combination of genetic and environmental factors. Susceptibility genes, environmental triggers such as alterations in the microbiota, and epigenetic mechanisms all contribute to the development of GD and its extrathyroidal manifestations [20,21,22]. Human leukocyte antigen (HLA) complex variants contribute to 5% of the estimated genetic susceptibility and an association between TSH-R gene polymorphisms and GD risk has been described [22,23]. Several elements involved in the immune checkpoint and immune response regulation have also been reported to play a role. Indeed, polymorphisms of Forkhead box P3 (FOXP3), a crucial transcription factor involved in the development of regulatory T cells, and of FCRL3 protein, which contributes to regulating the immune system by affecting B-cell signaling, are associated with the risk of developing GD [24,25,26,27,28,29,30] (Table 1). Other immune-regulatory genes, including cluster of differentiation 40 (CD40), protein tyrosine phosphatase non-receptor type 22 (PTPN22), and cytotoxic T lymphocyte-associated antigen 4 (CTLA4), have been linked to GD [31,32] (Table 1).

CD40 plays a crucial role in B-cell activation through its interaction with the CD40 ligand (CD40L), which is expressed by activated CD4+ T cells [34]. The CD40–CD40L costimulatory pathway is a key contributor to the pathogenesis of GD, as it promotes the activation of autoreactive B cells. Additionally, the CD40 gene is recognized as a major susceptibility gene for GD [34]. Bufalo et al. conducted a case–control study confirming the significant role of CTLA4 polymorphisms in GD susceptibility and highlighting the influence of PTPN22 polymorphisms on the clinical manifestations of the disease, suggesting their potential impact on disease severity [23]. Over the past 15 years, one of the most significant paradigm shifts in B-cell biology has been the recognition that while B-cell receptor (BCR) expression is necessary for B-cell development and survival, it is not sufficient on its own. Instead, the B-cell activating factor (BAFF) has emerged as a crucial component for B-cell survival, without which B-cell maturation cannot occur. The elevated thyroidal expression of the BAFF and its receptor (BAFF-R) in patients with GD suggests their role in the disease’s pathophysiology [35,36,37,38].

In many autoimmune diseases, including GD, the genetic background can be modulated by both modifiable and non-modifiable environmental factors, with epigenetic mechanisms playing a central role. In GD, epigenetic alterations include hypermethylation at specific DNA sites in CD4+ and CD8+ T lymphocytes, histone modifications (e.g., acetylation and phosphorylation) associated with chromosomal abnormalities or other autoimmune conditions, and the regulatory effects of non-coding RNAs, such as microRNAs (miRNAs) [31,39]. MicroRNAs are a group of small, non-coding RNAs that regulate gene expression by binding to the 3′ untranslated region of their target mRNAs, leading to gene silencing [40]. MicroRNAs serve as key regulators of Toll-like receptor signaling, leading to the activation of NF-κB, IRF, and AP-1 transcription factors, which control the expression of pro-inflammatory cytokines [41]. However, their role in GD is still under debate. Over the past 10 years, the role of miRNAs in GD has been increasingly investigated. Indeed, serum levels of certain miRNAs, such as miR-181d, miR-16, miR-22, miR-375, and miR-451, are upregulated in GD patients compared to controls, while others, including miR-346 and miR-23a-3p, are downregulated [41,42]. Qin et al. reported that miR-22 and miR-183 were overexpressed in the thyroid tissue of GD patients, whereas miR-101, miR-197, and miR-660 levels were downregulated [43]. Interestingly, Martínez-Hernández et al. indicated that miR-21-5p, miR-96-5p, miR-Let7d-5p, miR-142-3p, and miR-301a-3p were positively correlated with TRAb levels and a higher severity of the disease, defined by higher recurrence rates and the presence of active GO [44]. Regarding the mechanisms through which miRNAs may contribute to the development of GD, experiments performed in animal models have demonstrated that miR-23a-3p overexpression enhances Treg function in vivo [26]. However, further studies are needed to elucidate the role of miRNAs in GD and determine whether they may serve as biomarkers for its diagnosis, treatment, and prognosis.

Psychological stress, iodine excess, irradiation, the post-partum period, and viral infections have been recognized as environmental risk factors for GD [7,20]. Among viral infections, severe acute respiratory syndrome coronavirus 2 (SARS-CoV-2) has been hypothesized to contribute to GD development through various mechanisms, though further studies are needed to confirm this association [45,46]. Interestingly, immune-modulating agents, such as alemtuzumab, have also been reported as potential triggers [47]. New evidence indicates that the gut microbiota exerts an important role in GD development [41]. An analysis of bacterial diversity revealed that patients with GD have reduced gut microbial diversity compared to healthy controls. The gut microbiota in GD patients predominantly consists of four phyla: Firmicutes, Bacteroidetes, Proteobacteria, and Actinobacteria. Several studies have consistently shown that GD patients exhibit increased abundances of Bacteroidetes and Proteobacteria and decreased levels of Firmicutes compared to controls [48]. Similarly, another study identified an increased relative abundance of Bacteroidetes and Actinobacteria and a reduction in Firmicutes in GD patients [49].

The interplay between genetic and epigenetic factors may influence various stages of GD pathogenesis, contributing to the loss of immune tolerance and increasing individual susceptibility to developing the disease.

## 3. Conventional Treatments

Current treatment options for GD are primarily aimed at managing hyperthyroidism. However, none of these approaches address the underlying pathogenic processes of the condition and are not without limitations. Antithyroid drugs (ATDs) are associated with a considerable risk of hyperthyroidism recurrence, while radioactive iodine (RAI) therapy and thyroidectomy lead to permanent hypothyroidism [50,51].

ATDs are the first line treatment for GD [52]. Three compounds are available, with methimazole (MMI) as the classical and most widely distributed ATD, while carbimazole (CBM) is an inactive drug metabolized to MMI in the blood with a lower potency than MMI [53]. Propylthiouracil (PTU) is 10-fold weaker than MMI, while it additionally inhibits desiodase type 1, the enzyme responsible for converting thyroxine (T4) to 3.5.3′-triiodothyronine (T3) [54]. The initial dose of MMI is usually 10–30 mg daily depending on the severity of hyperthyroidism while PTU is given at a dose of 150–300 mg daily [55]. The usual daily maintenance doses of ATDs in the titration regimen are 2.5–10 mg of MMI and 50–100 mg of PTU [55]. According to the ATA and ETA guidelines, ATDs are usually administered for 12–18 months. However, over the past two decades, numerous studies have demonstrated that long-term antithyroid treatment is both effective and safe for maintaining euthyroidism. Furthermore, it has been observed that serum TRAb levels may not permanently decrease until after 5 to 6 years of ATD treatment and that long-term therapy with ATD is associated with a higher remission rate [56].

ATDs are associated with a range of adverse events (AEs), which can be categorized as minor, such as rash (3–6%), pruritus (2–3%), urticaria (1–2%), dyspepsia (3–4%), nausea (2.4%), and arthralgia (1.6%), or major, namely agranulocytosis, hepatotoxicity, vasculitis, and, pancreatitis [57]. Only about 50% of patients receiving ATDs for 12 to 18 months will achieve a permanent remission [55,58]. RAI and thyroidectomy may be proposed to GD patients in these cases, as well as patients experiencing a recurrence or relapse of thyrotoxicosis, and those with poor adherence to ATD treatment or an occurrence of significant ATD-related side effects [8]. Although RAI may cause hypothyroidism, necessitating lifelong replacement with levothyroxine, it is a safe, well-tolerated, and long-term effective option for GD. Thyroidectomy is a viable option in specific cases, including patients with large goiters, uncontrolled hyperthyroidism, moderate-to-severe active GO, suspected thyroid cancer, or those who express a preference for surgical intervention [59]. Proper preparation with ATDs is essential before thyroidectomy, particularly in cases of severe hyperthyroidism, to prevent perioperative complications and ensure stable thyroid function [55,60]. While potential complications include laryngeal edema, recurrent laryngeal nerve injury, hypocalcemia, bleeding, and hypoparathyroidism, these risks are notably low when the surgery is conducted by experienced, high-volume surgeons [61,62]. Postoperatively, levothyroxine replacement therapy is initiated using a dosage calculated based on the patient’s body weight [9].

## 4. Novel Therapeutic Approaches

**Figure 1:**
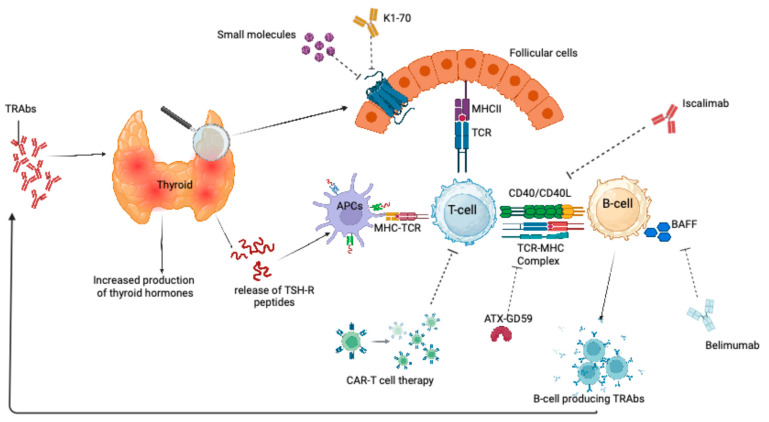
Mechanisms of action of emerging therapies for Graves’ disease.

Recent in vitro and in vivo research aims to introduce and establish new treatments for the management of GD. Innovative treatments, including drugs targeting CD20, CD40-CD40L signaling, BAFF, FcRn, the HLA-DR3 pathway, and TSH receptor signaling, are currently under investigation (Figure 1).

The breakdown of self-tolerance leads to the recognition of thyroid-stimulating hormone receptor (TSH-R) peptides by antigen-presenting cells (APCs). T-cell receptors (TCRs) recognize the TSH-R peptides, and the interaction between TCR and the major histocompatibility complex (MHC) triggers T-cell activation. Activated T cells stimulate B cells through TCR–MHC interactions and CD40–CD40L binding. Upon activation, B cells differentiate into plasma cells that produce TSH-R autoantibodies (TRAbs), which stimulate thyroid cell proliferation and hormone secretion, ultimately leading to hyperthyroidism. The figure illustrates therapeutic targets of recently proposed treatments for Graves’ disease (GD). K1-70 is a human monoclonal antibody that binds to and blocks TSH-R. Iscalimab, an anti-CD40 monoclonal antibody, inhibits CD40–CD40L co-stimulatory signaling. ATX-GD-59 is an apitope therapy designed to prevent TSHR autoantibody production and restore immune tolerance. Belimumab, a fully human monoclonal antibody targeting the B-cell-activating factor (BAFF), blocks the BAFF from interacting with its receptors, thereby inhibiting primary humoral immune responses and depleting BAFF-dependent naïve B cells. Additionally, small molecules targeting the transmembrane domain of TSH-R prevent receptor activation, while autoantigen-based Chimeric antigen receptor (CAR) T cells can directly target and kill auto-reactive B lymphocytes through targeted recognition.

### 4.1. Anti-CD20 Monoclonal Antibody—Rituximab

Rituximab (RTX) is an anti-CD20 monoclonal antibody (mAb) that depletes B-cells via complement-mediated cytotoxicity, antibody-dependent cellular cytotoxicity and direct apoptosis [63]. While RTX is currently used as a second line treatment for moderate-to-severe, active GO [64], its efficacy in GD is still under investigation. A small prospective study published in 2008 observed a beneficial effect of RTX in mild relapsing GD [65]. The RIGD study, a multicenter phase 2 clinical trial, evaluated RTX treatment in 27 young patients (12–20 years old) with GD. Participants received a single 500 mg dose of RTX along with ATDs for 12 months. After 2 years, 13/27 (48%) patients achieved hyperthyroidism remission, indicating that the combination of RTX with an antithyroid drug may increase the likelihood of remission in GD patients [66]. Another prospective study compared short-term methimazole therapy with or without RTX in 20 GD patients, showing a 40% remission rate after 24 months of follow-up in those treated with RTX. However, RTX was most successful in patients with TRAb levels < 5 UI/L at presentation [67]. Some studies have reported a reduction in serum TRAb levels following RTX therapy, although this was not consistent across all studies [65,67,68,69]. The most common side effects of RTX observed in clinical trials for GD and GO are mild infusion-related reactions, such as throat itching, nasal stuffiness, nausea, and slight temperature elevation [65,66,67,68,69,70]. Articular adverse events and ulcerative colitis were described only in patients who received RTX without concurrent immunosuppression [71]. Progressive multifocal leukoencephalopathy and vasculitis have also been described [64,72]. In summary, evidence from small RCTs is insufficient, and given the side effects, larger multicenter RCTs are needed.

### 4.2. Anti-CD40 Monoclonal Antibody—Iscalimab

CD40, a member of the TNF receptor family, is expressed on antigen-presenting cells (APCs) and plays a pivotal role in immune responses. When CD40 binds to its ligand CD40L on T cells, it activates a co-stimulatory pathway that promotes B-cell proliferation, immunoglobulin class-switching, and germinal center formation [73]. Experimental studies in animal models have shown that blocking CD40 activation can suppress the development of GD [74], whereas the overexpression of CD40 in the thyroid enhances antibody production and exacerbates the severity of the disease [75]. Iscalimab is a human mAb that blocks CD40L from binding to CD40, inhibiting their costimulatory pathway and ultimately reducing B-cell activation and differentiation [76].

In an open-label trial, 15 adult patients with untreated Graves’ hyperthyroidism received five intravenous doses of iscalimab over a 12-week period [77]. Euthyroidism was achieved in 7/15 patients (47%) 24 weeks after the last dose. Among responders, 4/7 experienced a relapse. TRAb serum levels significantly decreased in all patients. Interestingly, all patients with TRAb < 20 IU/L were responders [77]. The observed adverse events, including fatigue, nausea, headache, insomnia, upper respiratory tract infection, and cystitis, were mild [77]. Overall, the efficacy of Iscalimab appears to be comparable to that of a 12-month course of ATDs, with a good safety profile. However, the small sample size limits the ability to draw definitive conclusions. To explore why approximately half of the patients did not respond to treatment, the potential role of genetic factors in predicting treatment outcomes has been performed, showing that specific genetic variants of the CD40 gene influence CD40 expression levels and are predictive of a favorable response to iscalimab treatment [34].

Beyond GD, iscalimab is under investigation for its potential therapeutic role in various immunological conditions, namely systemic lupus erythematosus, rheumatoid arthritis, myasthenia gravis, and solid organ transplantations [78].

### 4.3. Anti-BAFF Monoclonal Antibody—Belimumab

Belimumab, a humanized IgG1 monoclonal antibody specific to the BAFF, binds to the soluble BAFF and inhibits its biological activity. By blocking BAFFs, belimumab negatively regulates B cells, lowering their proliferation and survival and ultimately resulting in B-cell failure to differentiate into antibody-producing plasma cells [79].

Elevated circulating BAFF levels, along with a significant positive correlation between BAFFs and TRAbs, have been observed in patients with Graves’ hyperthyroidism [35]. Evidence supporting the involvement of BAFFs in the pathogenesis of GD includes their heightened expression in infiltrating immune cells and thyrocytes from affected individuals [36]. Moreover, studies in a mouse model of GD have shown that blocking the BAFF using a BAFF-specific receptor–Fc can mitigate hyperthyroidism [80]. These findings highlight the need for further clinical investigations to assess the potential of belimumab as a therapeutic option for GD.

### 4.4. Anti-FcRn Monoclonal Antibody—Batoclimab

Batoclimab is a human anti-neonatal Fc receptor (FcRn) mAb, which promotes autoantibody degradation, including that of TRAbs, by blocking FcRn-IgG interactions (30). The beneficial effect of batoclimab has been proven in several autoimmune conditions (e.g., myasthenia gravis and immune thrombocytopenic purpura) [81,82,83,84].

A recent proof-of-concept randomized clinical trial evaluated the efficacy of batoclimab in patients with Graves’ orbitopathy (GO) [85]. Participants received weekly subcutaneous injections of batoclimab at a dose of 680 mg for two weeks, followed by 340 mg for an additional four weeks. While the trial demonstrated a significant reduction in TRAb and total IgG serum levels, improvements in proptosis and Clinical Activity Scores (CASs) did not reach statistical significance at 12 weeks [85].

Although these findings remain preliminary, the mechanism of action of batoclimab and its potential applications in moderate-to-severe GO suggest that it may also be effective in managing Graves’ hyperthyroidism. Multicenter clinical trials are currently underway to explore its utility in controlling hyperthyroidism in patients with GD, and the results are eagerly anticipated.

### 4.5. TSHR-Blocking Antibodies—K1-70

From a patient with GD and TSHR-blocking antibodies, Evans et al. were able to clone a monoclonal TSHR-blocking antibody, known as K1-70 [86]. The treatment of the stimulating M22 antibody in rats resulted in a total suppression of the thyroid hormone increase [87]. The safety profile of K1-70 in GD patients was evaluated in a Phase I clinical trial. The expected effect occurred after a single IM dose of 25 mg or a single intravenous dose of 50 mg or 150 mg, shifting thyroid hormones and TSH values into the hypothyroid range. A clinically significant improvement in symptoms of both GD and GO was also observed. K1-70TM was well tolerated by all subjects at all doses, and no significant immunogenic events were observed [88].

### 4.6. Small Molecule TSHR Antagonists—ANTAG-3, VA-K-14 and S37

Numerous small molecules have been developed to function as inverse agonists of the TSH receptor (TSHR), effectively inhibiting both basal and agonist-induced signaling. Among the most extensively studied are ANTAG-3, VA-K-14, and S37, which interact with TSHR at sites that are distinct from the extracellular domain where TSH and TRAb bind. These compounds have shown promise in preventing TRAb-induced Graves’ hyperthyroidism by reducing thyroid hormone levels in animal models and inhibiting TSH-stimulated cAMP synthesis in vitro [89,90,91]. In mice treated with TRH, ANTAG-3 led to a 44% reduction in serum free T4 levels and significantly decreased the expression of sodium-iodide cotransporter and thyroperoxidase mRNAs by 75% and 83%, respectively [88]. VA-K-14 demonstrated the ability to inhibit the TSHR stimulation induced by sera from GD patients and monoclonal-stimulating TSHR antibodies [92]. S37 was identified as an effective antagonist that suppressed thyrotropin-induced cyclic adenosine monophosphate accumulation in HEK 293 cells expressing TSHR, while notably sparing the closely related follitropin and lutropin receptors. Given the structural homology among TSHR, the follicle-stimulating hormone receptor, and the luteinizing hormone/chorionic gonadotropin receptor, potential off-target reproductive effects remain a concern [91]. Although none of these compounds have been tested in humans to date, this approach holds promise for the management of hyperthyroidism.

### 4.7. DRβ1-Arg74 Blocker—Cepharantine

Cepharanthine is a plant-derived alkaloid extracted from Stephania cepharantha Hayata. For nearly four decades, it has been used in Japan to treat a range of acute and chronic conditions, including alopecia areata, radiation-induced leukopenia, and thrombocytopenic purpura associated with multiple myeloma. While no significant adverse effects have been reported thus far, larger clinical trials are needed to fully assess its safety profile, as only a limited number of studies have been conducted to date [93]. Cepharanthine has been shown to inhibit antigen presentation by suppressing dendritic cell activation and function [94]. Li, Tomer, and colleagues previously demonstrated that Cepharanthine blocks peptides from binding to HLA-DRβ1-Arg74 and attenuates T-cell responses to thyroglobulin (Tg) in an experimental autoimmune model of Hashimoto’s thyroiditis induced in NOD-DR3 mice [95]. Using virtual screening, ELISA, and cellular binding assays, they identified a high-affinity TSHR peptide, called TSHR.132, that binds to HLA-DRβ1-Arg74. To further investigate its role in GD, they induced experimental autoimmune GD (EAGD) in a novel BALB/c-DR3 humanized mouse model through cDNA immunization with an adenovirus expressing the TSHR A-subunit. Their findings confirmed that TSHR.132 is a key DRβ1-Arg74 binding peptide that triggers GD and demonstrated that Cepharanthine reduces its presentation and the corresponding T-cell response in the EAGD model [96]. Targeting the HLA-DRβ1-Arg74 pocket with compounds like Cepharanthine offers a personalized medicine approach for autoimmune thyroid diseases (AITDs), as treatment would be tailored specifically to individuals carrying the HLA-DRβ1-Arg74 risk variant. These findings open new avenues for the development of innovative therapies for GD.

### 4.8. Immune Tolerance—ATX-GD-59

Antigen-specific immunotherapy, which involves the gradual administration of small amounts of antigens, is well established for restoring immune tolerance in allergies. However, in autoimmune diseases, this approach has been complicated by immune responses to the administered antigens [97]. This risk can be mitigated through tolerance induction strategies, taking advantage of synthetic antigen-processing independent epitopes (apitopes) that have processed CD4+ T-cell epitopes [98]. ATX-GD-59 is a peptide-based immunotherapy composed of two soluble synthetic peptides derived from the human TSHR sequence. ATX-GD-59 functions as an apitope by binding with high affinity to HLA-DR molecules on the surface of immature dendritic cells in regional lymph nodes, thereby reducing the activation of antigen-presenting cells [99]. In an open-label phase I study, ten previously untreated patients with mild to moderate Graves’ hyperthyroidism received ten intradermal doses of ATX-GD-59 over 18 weeks. Among them, five (50%) achieved free triiodothyronine levels within the reference range, and serum TRAb concentrations decreased over the course of the study. The most commonly reported adverse events were mild swelling and pain at the injection site [100]. Given its ability to modulate the immune response without causing generalized immunosuppression, ATX-GD-59 represents a promising therapeutic option for GD.

### 4.9. Chimeric Antigen Receptor (CAR) T-Cell Therapy

Chimeric antigen receptor (CAR) T-cell therapy has emerged as a transformative treatment in oncology, leveraging engineered T cells to selectively target pathogenic cells [101]. This approach offers a unique advantage by specifically eliminating pathogenic autoimmune cells while promoting protective immunity, making it an attractive therapeutic option for autoimmune diseases. Autoantigen-based CAR-T cells can directly target and kill auto-reactive B lymphocytes through targeted recognition. Notably, CAR-T-cell therapy surpasses monoclonal antibodies, like rituximab, due to its ability to generate migration to lymphatic nodules, fostering the development of effective and memory cells with just a single application [102]. Consequently, CAR-T cells hold the potential to outperform monoclonal antibody therapy in terms of specific targeting, reduced adverse effects, and a possible contribution to the permanent restoration of immune balance [103,104].

Interestingly, recent studies have demonstrated the potential of TSHR-based CAR-T cells to specifically deplete TRAb-producing B cells, offering a promising therapeutic approach for GD [105]. Indeed, a novel TSHR-CAR-T incorporating the extracellular domain of the TSH receptor fused with the CD8 transmembrane and intracellular signal domain has been engineered. TSHR-CAR-T cells demonstrated the ability to recognize and effectively eliminate TRAb-producing B lymphocytes both in vitro and in vivo [105]. These findings suggest that TSHR-CAR-T cells offer a promising and innovative immunotherapeutic approach for the treatment of GD and related conditions.

Despite these promising results, several challenges must be addressed before CAR-T therapy can be widely adopted for autoimmune diseases. One major concern is the potential for off-target effects, which can lead to unintended immune suppression or tissue damage. Additionally, the durability of CAR-T-cell responses in the autoimmune setting remains uncertain, as prolonged immune modulation could increase the risk of infections or secondary autoimmune disorders. Another challenge is the potential for cytokine release syndrome that could pose significant safety concerns in autoimmune patients. Furthermore, the high cost and complexity of CAR-T production could limit accessibility. Addressing these challenges will be essential to define the therapeutic potential of CAR-T-cell therapy in autoimmune diseases.

## 5. Conclusions

Several treatments are currently available to manage GD, though most do not directly target its underlying immunopathogenic mechanisms. Treatment selection should be based on the latest international guidelines and tailored to each patient’s clinical profile. ATDs remain the first-line therapy but carry a high relapse rate after discontinuation. RAI ablation and total thyroidectomy are a valid, widely accepted, cost-effective, and safe option offering permanent solutions but require lifelong levothyroxine replacement and pose risks associated with radiation exposure, surgery, and anesthesia.

Advancements in research are paving the way for new therapeutic strategies that may transform the treatment paradigm. Emerging approaches include restoring immune tolerance through immunomodulatory TSHR peptides and targeting TSHR signaling via rituximab, iscalimab, and belimumab. Moreover, inhibiting IgG recycling through the neonatal Fc receptor blockade—an approach already explored in other autoimmune diseases—may hold promise for GD. However, most clinical studies on these novel treatments are still in early phases, predominantly open-label phase I and II trials. To confirm their efficacy and safety, further randomized controlled trials are essential, offering hope for more targeted and personalized management strategies which can be introduced in our clinical practice in the future. Notably, while these therapies offer exciting prospects for more targeted and personalized management, their widespread clinical implementation may be challenged by high costs and logistical complexities. Biologic agents and advanced immunotherapies often require specialized manufacturing, storage, and administration, which may limit accessibility, particularly in resource-limited settings. Additionally, their long-term cost-effectiveness remains uncertain, necessitating further studies to assess their feasibility for routine clinical use.

## Figures and Tables

**Table 1:** Genetic factors involved in Graves’ disease (GD) development.

Gene	Protein Localization	Target	Main Function	Genetic and Functional Changes in GD
*CTLA-4* [33]	T-cell membrane	Co-stimulatory molecule (e.g., CD80, CD86) on APCs.	Negative regulation of the immune response, acting as a ‘brake’ for activated T cells.	Polymorphisms associated with reduced inhibitory function, leading to increased T-cell activation and heightened GD risk.
*PTPN22* [33]	T-cell cytoplasm	Tyrosine kinases (e.g., Lck, ZAP-70) involved in T-cell receptor (TCR) signaling.	Dephosphorylation of tyrosine kinases involved in the regulation of immune response.	Polymorphisms that affect enzyme activity, resulting in increased T-cell activation and higher risk of GD.
*FCRL3* [2,8,30]	B-cell membrane	Several ligands, including FcγRIIb.	Negative regulation of B-cell proliferation and differentiation.	Functional alterations leading to enhanced B-cell activation and increased production of TRAbs.
*CD40* [33,34]	APC and B-cell membrane	CD40L (also called CD154) on activated T cells.	Stimulation of T-cell activation and B-cell maturation.	Overexpression and/or hyperactivation contributing to excessive immune responses and TRAbs’ production.
*FOXP3* [2,4,27]	T-cell cytoplasm and nucleus	Regulatory pathways in T cells.	Critical for maintaining immune tolerance by regulating Treg activity.	Polymorphisms and abnormal acetylation associated with reduced Treg activity, resulting in an exaggerated immune response and increased GD susceptibility.

GD, Graves’ disease; CTLA-4, cytotoxic T lymphocyte antigen-4; APCs, antigen-presenting cells; PTPN22, protein tyrosine phosphatase non-receptor type 22; FCRL3, Fc receptor-like protein 3; TRAbs, thyrotropin receptor antibodies; CD40, cluster of differentiation 40; CD40L, CD40 ligand; FOXP3, Forkhead box P3; Treg, regulatory T cells.
